# Early Implant Placement with Immediate Loading in the Mandibular Anterior Region: A Rapid Solution to Edentulism

**DOI:** 10.1155/2023/8487094

**Published:** 2023-12-18

**Authors:** Arjun Hari Rijal, Bhageshwar Dhami, Pratistha Ghimire, Manoj Humagain, Simant Lamichhane

**Affiliations:** ^1^Department of Periodontology and Oral Implantology, Kathmandu University School of Medical Sciences, Dhulikhel, Kavrepalanchok, Nepal; ^2^Department of Periodontology and Oral Implantology, Kantipur Dental College and Hospital, Basundhara, Kathmandu, Nepal; ^3^Dental Department, Methinkot Hospital, Kavrepalanchok, Nepal

## Abstract

The aim of this article is to present the case of an early implant placement with immediate loading in the mandibular anterior region as a rapid solution to edentulism. A 40-year-old healthy male patient reported with a chief complaint of loosening of tooth in the lower front region of the jaw. On intraoral examination, there was a mobile tooth with respect to 41. The mobile tooth was extracted, and early implant placement was done along with Bio-Oss bone grafts to fill the jumping distance with no barrier membrane. Immediate provisionalisation was done on early-placed dental implants. After 5 months of the healing period, the final implant-level impressions were made, and the provisional crown was replaced with the final zirconia crown. This case report demonstrates satisfactory esthetic and functional outcomes along with various other advantages.

## 1. Introduction

Brånemark et al. [1] introduced the classic protocol for dental implant therapy according to which postextraction healing period of at least 6 months was allowed before implant placement. Since complete soft and hard tissue healing after tooth extraction is required to achieve successful osseointegration, classic protocol was followed [1, 2]. In the present time, esthetic outcome is influenced by the timing of implant placement postextraction in the esthetic zone [3]. The clinician has four different treatment options, as defined by two ITI Consensus Conferences 2003 and 2008 [3, 4]. One of these options is early implant placement in which implants are placed after 4 to 8 weeks of soft-tissue healing [5].

Early implant placement is a viable treatment alternative in which implant is placed following complete soft tissue coverage of the extraction socket [4]. This allows for the resolution of local pathology and provides enhanced soft tissue volume [6, 7]. According to various studies, clinical outcomes for implants placed following early placement protocol are promising [7–10].

According to Anibali et al., early placement can be regarded as an appropriate alternative to immediate placement when clinical outcome of immediate implant placement could be affected by unfavourable conditions at the time of extraction [11]. When compared with the delayed implant placement protocol, early dental implant placement offers advantages in the preservation of soft and hard tissues [12].

This case report showcases a technique involving early implant placement with immediate loading in the mandibular anterior region, offering a prompt and effective approach to address edentulism while preventing any potential loss of hard and soft tissues. This method also ensures optimal esthetics and functionality, providing a swift solution to the issue.

## 2. Case Presentation

A 40-year-old male patient reported to the Department of Periodontology, Kantipur Dental College, Kathmandu, Nepal, with a chief complaint of loosening of tooth in the lower front region of the jaw.

On taking history, the patient had mobile tooth in the lower front region of the jaw for 6 months. There was no significant medical history of the patient. During the intraoral examination, it was observed that the patient had good oral hygiene, mobility in tooth 41, and a satisfactory amount of keratinized tissue surrounding the missing tooth. An intraoral periapical radiograph was advised which revealed periapical lesion w.r.t 41. An array of treatment options was discussed with the patient for the replacement of the missing tooth after extraction. A detailed explanation of the benefits and drawbacks of each treatment option was provided to the patient, after which he chose the dental implant therapy.

**Figure 1:**
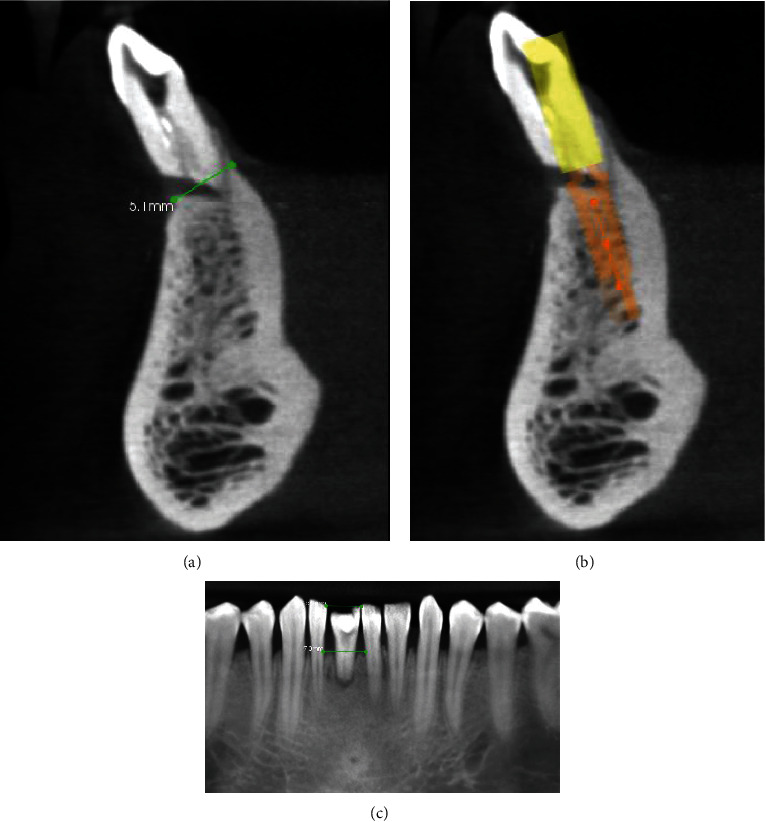
(a, b) Cone-beam computed tomography (CBCT) in sagittal section with dental implant planning with respect to 41. (c) Mesiodistal measurement at the proposed implant site.

Cone-beam computed tomography (CBCT) was indicated for the patient, and the report revealed sufficient mesiodistal and buccolingual width, as well as a safe distance from vital structures (Figures 1(a)–1(c)). CBCT revealed mesiodistal dimension at bone crest level and incisal edge as 7 mm and 5.5 mm, respectively. Similarly, there was adequate buccolingual width of 5.1 mm present w.r.t. 41. Consequently, the recommended course of action involved tooth extraction, followed by early implant placement. The patient was referred to the Department of Oral Surgery for atraumatic extraction, during which granulation tissues were meticulously removed using a bone curette. Subsequently, the patient's progress was monitored through regular follow-up appointments.

**Figure 2:**
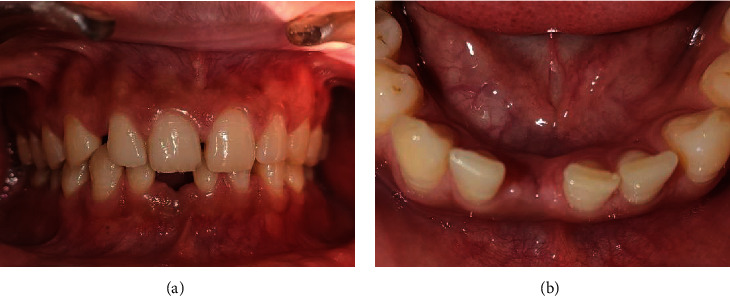
(a, b) Preoperative view of the partially edentulous site with respect to 41.

**Figure 3:**
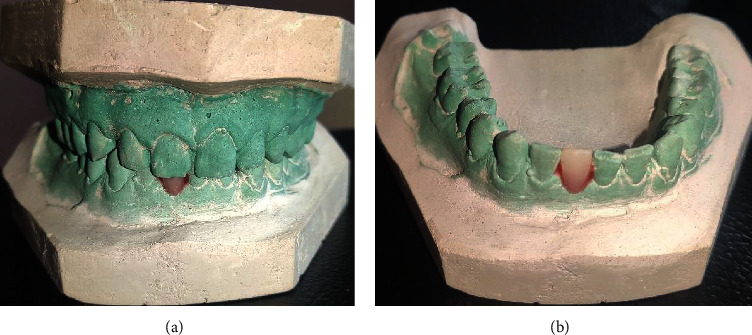
(a, b) Diagnostic cast with mock-up using acrylic tooth w.r.t 41.

At 4 weeks postextraction, the intraoral examination revealed good soft tissue healing with respect to 41 (Figures 2(a) and 2(b)). At the same appointment, a diagnostic impression was made with irreversible hydrocolloid and poured in dental stone. The obtained cast was used for mock-up using acrylic tooth w.r.t 41 (Figures 3(a) and 3(b)). After evaluating the CBCT results, 3.0 × 11 mm of implant size (NobelReplace® Conical Connection, Nobel Biocare Services AG, P.O. Box, CH-8058 Zürich-Flughafen Switzerland) was planned for the patient.

**Figure 4:**
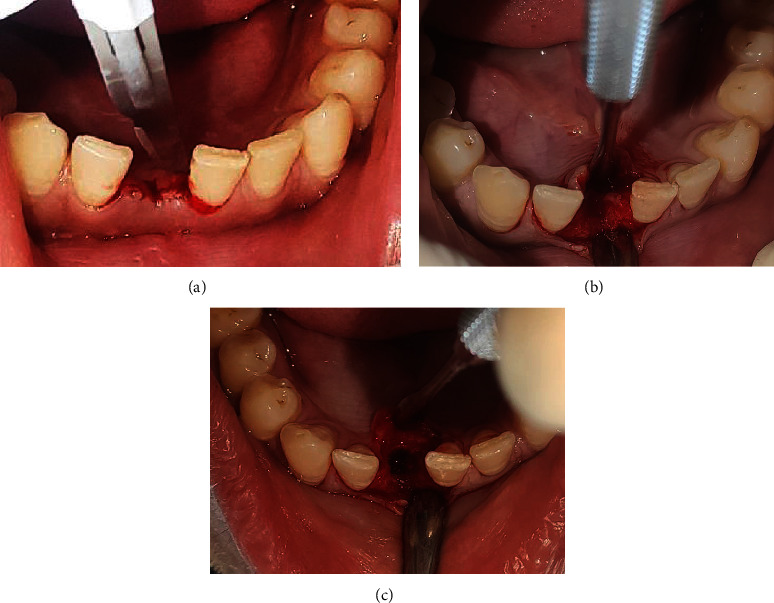
(a) Midcrestal incision, (b) full-thickness mucoperiosteal flap reflection, and (c) preparation of osteotomy site.

**Figure 5:**
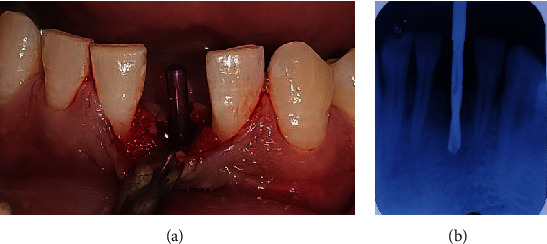
(a) Paralleling pin placement. (b) Intraoral periapical radiograph with guide pin.

**Figure 6:**
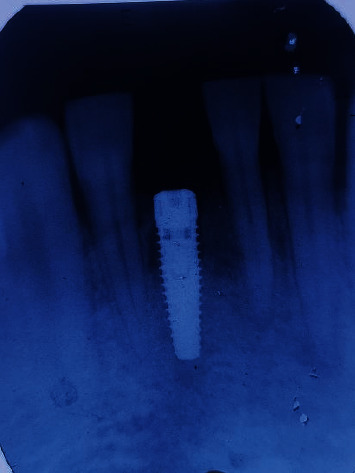
Postoperative intraoral periapical radiograph of dental implant.

After about 5 weeks of extraction, patient was recalled for early implant placement. We followed the standard surgical protocol for early implant placement by Buser et al. [7–10] in which their various publications were taken into consideration for better clinical outcomes. Informed as well as written consent was taken from the patient prior to the treatment. Anesthesia was achieved through the local infiltration of 2% Lignocaine HCl with 1 : 200,000 epinephrine. A midcrestal incision was performed using a number 15 BP blade (Figure 4(a)), and a full-thickness mucoperiosteal flap was gently raised to visualize the available alveolar bone in both the mesiodistal and buccolingual directions (Figure 4(b)). The soft tissue remnants from the implant surgical site were cleared and irrigated with normal saline. Subsequently, the osteotomy site preparation was performed with respect to tooth 41 (Figure 4(c)). Initial pilot drill of 2.0 mm from the implant surgical kit (Nobel Biocare Services AG, P.O. Box, CH-8058 Zürich-Flughafen Switzerland) was used as the first drill. Next, a guide pin was placed into the osteotomy site to ensure its alignment parallel to the adjacent tooth (Figure 5(a)) which was confirmed with intraoral periapical radiograph (Figure 5(b)). Final implant placement was then done, and again, intraoral periapical radiograph was taken (Figure 6).

**Figure 7:**
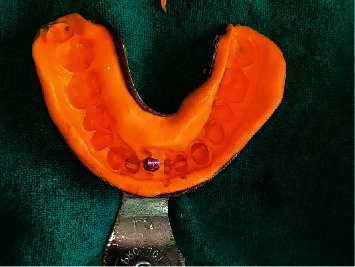
Closed tray impression for provisional crown.

**Figure 8:**
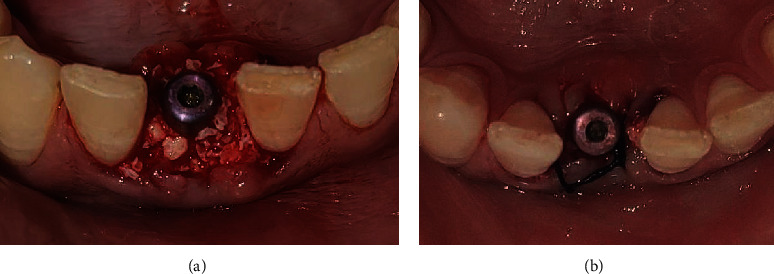
(a) Bone graft placement. (b) Suturing with 3-0 silk sutures.

**Figure 9:**
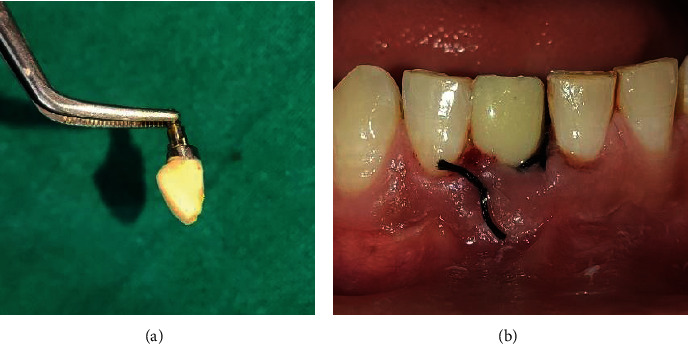
(a, b) Provisional crown and immediate loading w.r.t 41.

**Figure 10:**
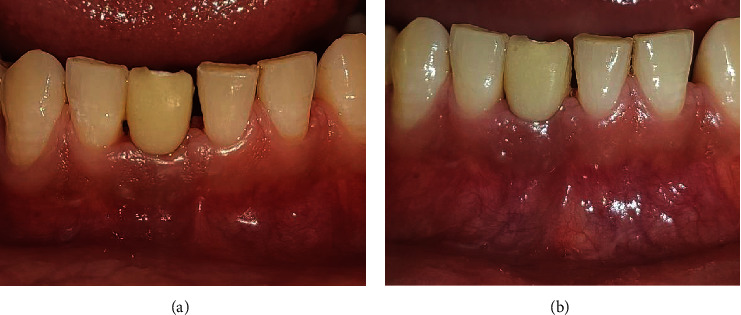
(a) Postoperative 2-month follow-up. (b) Postoperative 3-month follow-up.

**Figure 11:**
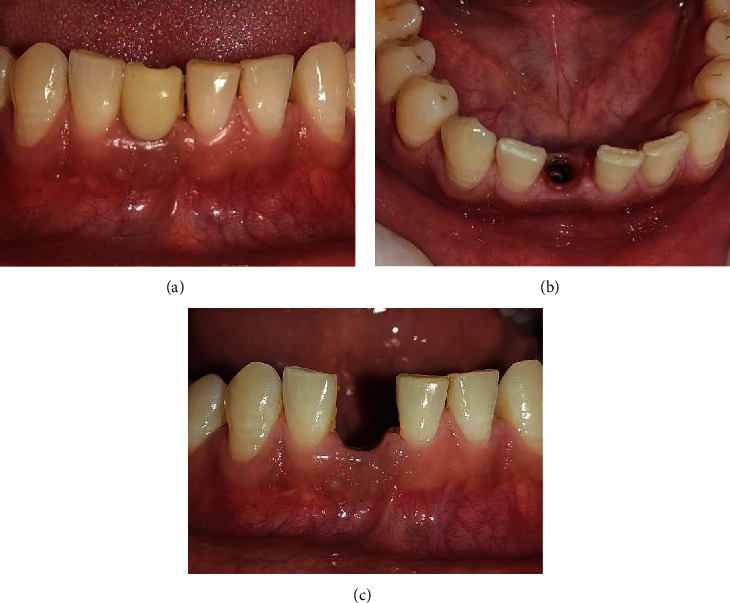
(a–c) Postoperative 5-month follow-up in different views.

With temporary abutment in place, closed tray impression was made using putty and light body (Figure 7) and sent to the lab for provisional crown fabrication. Healing cap was screwed into the implant. Jumping distance was filled with Xenografts (Geistlich Bio-Oss®, Geistlich Pharma AG, Bahnhofstrasse 40, CH-6110 Wolhusen, Switzerland) (Figure 8(b)), and two interrupted 3-0 silk sutures (ETHILON®, Ethicon Inc., Johnson & Johnson, Piscataway, NJ, USA) were placed w.r.t 41 (Figure 8(b)). The patient was well informed about the possible risks associated with implant surgery. Mouthwash with 0.2% chlorhexidine gluconate (CHX oral rinse—100 ml mouthwash) 10 ml twice daily for 21 days was advised. Nonsteroidal anti-inflammatory drugs (NSAIDs) (ibuprofen 400 mg and paracetamol 375 mg, Flexon tablet, Aristo Pharmaceuticals Pvt., Ltd.) (23-A, Shah Industrial Estate, Off Veera Desai Road, Andheri (West), Mumbai, 400053, India) as per need and antibiotics (Clavam 625 mg tablets, amoxicillin 500 mg+clavulanic acid 125 mg, Chennai, Tamil Nadu, India) three times a day for five days were prescribed to the patient. After provisional crown fabrication, occlusal adjustments were done so that no contact is established with opposing teeth, and immediate loading was done (Figures 9(a) and 9(b)). After one week following surgery, the patient was recalled for suture removal and evaluation of the surgical site. Regular follow-up visits at 2 and 3 months were scheduled for the patient (Figures 10(a) and 10(b)). On reevaluation during follow-up visits, soft tissue healing was found to be satisfactory. The patient was then recalled after five months for the final prosthesis (Figures 11(a)–11(c)).

**Figure 12:**
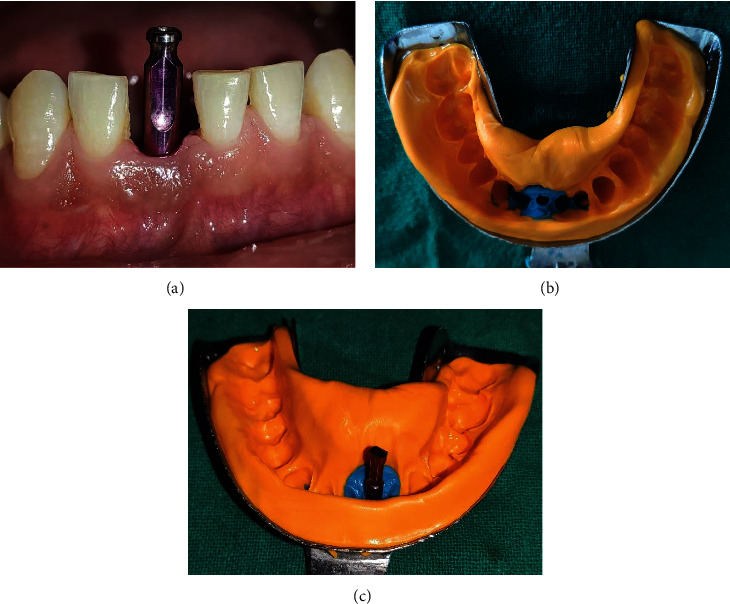
(a) Impression coping connected to the implant fixture. (b) Final impression with close tray technique. (c) Implant analogue attached to impression coping.

**Figure 13:**
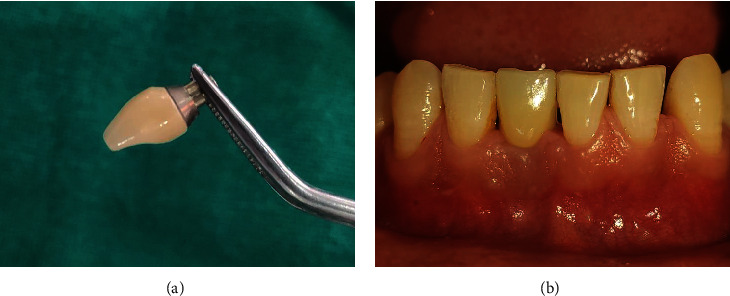
(a, b) Final prosthesis and its placement w.r.t 41.

At 5 months, the temporary crown was removed, impression coping was connected to the implant fixture (Figure 12(a)), and the final impression was made using putty and light body using a closed tray technique (Figure 12(b)). The implant analogue was then attached to the impression coping (Figure 12(c)). Shade selection was done, and the impression was sent to the lab for the final zirconia prosthesis. After occlusal adjustments, the final prosthesis was cemented w.r.t 41 using type II GIC as a luting agent (Figures 13(a) and 13(b)). The patient was kept on regular follow-up visits.

## 3. Discussion

Implant placement and loading protocols are considered as key elements in implant treatment planning. In the past, implant therapy was typically conducted in fully healed areas of patients with complete tooth loss [13]. But this conventional therapy was not accepted well by the clinician as well as the patient because of increased treatment time. Furthermore, achieving favorable esthetics was particularly challenging due to the alveolar bone remodeling that occurs after tooth extraction [13]. In 2003 and 2008, the International Team for Implantology issued comprehensive guidelines regarding the timing of implant placement [3, 4]. These guidelines encompassed various approaches: immediate implants placed on the same day of extraction, early implants with soft tissue healing between 4 and 8 weeks after extraction, early implants with partial bone healing between 12 and 16 weeks after extraction, and delayed implants placed after 6 months following extraction [3, 4].

According to studies, immediate and early implant placement in the single edentulous site in the anterior maxilla showed similar results in terms of ridge dimensional changes and acceptable clinical, aesthetic, and patient-reported outcomes [14]. However, in comparison to early implant placement, immediate implants were found to have a higher incidence of midfacial recession, affecting approximately 26% of cases [15]. Other studies also reported an incidence of significantly increased midfacial mucosal recession with immediate implants (0.85 mm) when compared with early implants (0.06 mm) [16].

Early implant placement has been associated with a significant increase, reportedly up to sevenfold, in soft tissue thickness, especially in cases involving thin or damaged facial walls. This increase results in the formation of a thick mucoperiosteal flap, promoting better vascularity for improved healing. Furthermore, the likelihood of flap tears and the need for soft tissue graft augmentation are reduced as well [17]. Opting for early implant placement with soft tissue healing also offers the advantage of obtaining an additional 2-3 mm of keratinized mucosa surrounding the implant. This benefit is attributed to the natural process of spontaneous soft tissue healing and apical bone formation that occurs during this approach [13]. Studies have reported stable soft tissue conditions in early implant cases after more than 3 years [7].

Immediate loading is defined as a prosthesis being placed in occlusion within 48 hours of implant surgery [18] or after 72 hours of implant placement [19]. The survival rate of implants as well as marginal bone loss was not affected by the difference between immediate and early loading at 1 or 3 years. So, either the immediate or early loading of the implants should be considered [20]. But patients always prefer to be rehabilitated as soon as possible, provided there is less risk of implant failure [20].

The use of xenograft for reduced dimensional alterations of a postextraction site was described in animal studies [21, 22]. The use of Bio-Oss® Collagen graft to fill the buccal gap after implant placement has been found to reduce the buccal vertical resorption from 1.3 ± 0.7 mm to 0.1 ± 0.5 mm [22].

Several clinical studies demonstrate the use of xenograft and resorbable barrier membrane in conjunction with early implant placement with successful results [7, 23, 24].

In contrast to these studies, the rationale for bone augmentation with early implant placement was different in the presented case. In this case, the jumping gap was filled with xenograft materials (Bio-Oss), and no barrier membranes was used. Here, no barrier membrane resulted in reduced surgical time and cost and also enhanced bone regeneration since the periosteum was not isolated from the grafted site. The periosteum has a vital role in bone graft incorporation, healing, and remodeling since it has multipotent mesenchymal stem cells which are the sources of blood vessels and growth factors, capable of differentiating into bone and cartilage [25].

The case presented demonstrated outstanding functional and aesthetic results, showcasing the notable benefits of early implant placement with soft tissue healing. These benefits encompassed the lack of soft tissue recession, no vertical bone loss observed six months postimplant placement, and the presence of an ample soft tissue barrier surrounding the implant in all directions. Both extraoral and intraoral structures play a crucial role in meeting the esthetic needs of patients [26, 27]. Specifically, early implant placement is essential for maintaining the intraoral soft tissue condition.

There are few limitations of early implant placement like more surgical time required, multiple surgical procedures starting from extraction to implant placement, and more invasive as compared to immediate implant placement [28]. Similarly, the survival rate of early implant placement with immediate loading (first-year survival rate = 98.3%) is slightly higher as compared to immediate implant placement with immediate loading (first-year survival rate = 94.6%) [29].

## 4. Conclusion

There are four different treatment options available for clinicians for postextraction implant placement. In the anterior region, the esthetic outcome and its long-term esthetic stability are of paramount importance which is the most important goal of implant therapy followed by proper function and phonetics. Early implant placement with soft tissue healing allows for 4-8 weeks of healing period following extraction before implants are placed (Table 1). This is used when the facial bone wall is thin or damaged and local bone anatomy allows proper 3D implant position providing good primary stability. This method averts the occurrence of vertical collapse near the implant sites, introduces keratinized tissue on all sides, and facilitates simpler apical engagement.

## Figures and Tables

**Table 1:** Summary of the research.

Key findings	Clinical implications
Periodontally compromised tooth with periapical pathology. No significant medical history.	Early implant placement with immediate loading was planned.
Mesiodistal dimension at crest of the bone radiographically: 7 mm Mesiodistal dimension at crest of the bone clinically: 8 mm Buccolingual width: 5.1 mm	3.0 × 11 mm of implant size (NobelReplace® Conical Connection) was selected.
Mesiodistal dimension: At height of contour: 5.5 mm Apicocoronal height: 10 mm	Adequate height and width for crown placement.
More than 35 Ncm torque was achieved.	Immediate loading was possible within 72 hours.
Limitation of the present study	1. At the time of tooth extraction, cone-beam computed tomography (CBCT) was performed. There may have been a certain amount of bone loss during the four-week interval. Repeating the CBCT at the time of implant placement could accurately reproduce the extent of bone remodeling 2. The provisional restoration provided during immediate loading could have been enhanced to better maintain the papillary height 3. A long-term follow-up period is essential to accurately assess the success of the procedure. It is crucial for obtaining a proper conclusion regarding its overall success or failure
Recommendations	This case report documents early implant placement with immediate loading within a limited follow-up period. To draw meaningful conclusions, further research, such as randomized trials or longitudinal studies with larger sample sizes, is essential

## Data Availability

The datasets used during the treatment are available from the corresponding author on reasonable request.

## References

[1] Branemark P. I., Branemark P. I., Zarb G. A., Albrektsson T., Rosen H. M. (1985). Introduction to osseointegration. *Tissue-Integrated Prostheses: Osseointegration in Clinical Dentistry*.

[2] Adell R., Lekholm U., Rockler B., Branemark P. I. (1981). A 15-year study of osseointegrated implants in the treatment of the edentulous jaw. *International Journal of Oral Surgery*.

[3] Chen S. T., Buser D. (2009). Clinical and esthetic outcomes of implants placed in postextraction sites. *International Journal of Oral & Maxillofacial Implants*.

[4] Hämmerle C. H., Chen S. T., Wilson T. G. (2004). Consensus statements and recommended clinical procedures regarding the placement of implants in extraction sockets. *International Journal of Oral & Maxillofacial Implants*.

[5] Buser D., Chen S. T., Weber H. P., Belser U. C. (2008). Early implant placement following single-tooth extraction in the esthetic zone: biologic rationale and surgical procedures. *International Journal of Periodontics & Restorative Dentistry.*.

[6] Chen S. T., Wilson T. G., Hammerle C. H. (2004). Immediate or early placement of implants following tooth extraction: review of biologic basis, clinical procedures, and outcomes. *International Journal of Oral & Maxillofacial Implants*.

[7] Buser D., Chappuis V., Bornstein M. M., Wittneben J. G., Frei M., Belser U. C. (2013). Long-term stability of contour augmentation with early implant placement following single tooth extraction in the esthetic zone: a prospective, cross-sectional study in 41 patients with a 5‐ to 9-year follow-up. *Journal of Periodontology*.

[8] Buser D., Halbritter S., Hart C. (2009). Early implant placement with simultaneous guided bone regeneration following single-tooth extraction in the esthetic zone: 12-month results of a prospective study with 20 consecutive patients. *Journal of Periodontology*.

[9] Buser D., Chappuis V., Kuchler U. (2013). Long-term stability of early implant placement with contour augmentation. *Journal of Dental Research*.

[10] Buser D., Wittneben J., Bornstein M. M., Grütter L., Chappuis V., Belser U. C. (2011). Stability of contour augmentation and esthetic outcomes of implant-supported single crowns in the esthetic zone: 3-year results of a prospective study with early implant placement postextraction. *Journal of Periodontology*.

[11] Annibali S., Bignozzi I., Iacovazzi L., La Monaca G., Paola C. M. (2011). Immediate, early, and late implant placement in first-molar sites: a retrospective case series. *International Journal of Oral and Maxillofacial Implants*.

[12] Sanz I., Garcia-Gargallo M., Herrera D., Martin C., Figuero E., Sanz M. (2012). Surgical protocols for early implant placement in post-extraction sockets: a systematic review. *Clinical Oral Implants Research*.

[13] Buser D., Chappuis V., Belser U. C., Chen S. (2017). Implant placement post extraction in esthetic single tooth sites: when immediate, when early, when late?. *Periodontology*.

[14] Arora H., Ivanovski S. (2018). Immediate and early implant placement in single-tooth gaps in the anterior maxilla: a prospective study on ridge dimensional, clinical, and aesthetic changes. *Clinical Oral Implants Research*.

[15] Chen S. T., Buser D. (2014). Esthetic outcomes following immediate and early implant placement in the anterior maxilla—a systematic review. *International Journal of Oral and Maxillofacial Implants*.

[16] Miyamoto Y., Obama T. (2011). Dental cone beam computed tomography analyses of postoperative labial bone thickness in maxillary anterior implants: comparing immediate and delayed implant placement. *International Journal of Periodontics and Restorative Dentistry*.

[17] Cochran D. L., Morton D., Weber H. P. (2004). Consensus statements and recommended clinical procedures regarding loading protocols for endosseous dental implants. *International Journal of Oral and Maxillofacial Implants*.

[18] Ibañez J. C., Jalbout Z. N. (2002). Immediate loading of osseotite implants: two-year results. *Implant Dentistry*.

[19] Pigozzo M. N., da Costa T. R., Sesma N., Laganá D. C. (2018). Immediate versus early loading of single dental implants: a systematic review and meta-analysis. *The Journal of Prosthetic Dentistry*.

[20] Araújo M., Linder E., Lindhe J. (2009). Effect of a xenograft on early bone formation in extraction sockets: an experimental study in dog. *Clinical Oral Implants Research*.

[21] Araújo M. G., Linder E., Lindhe J. (2011). Bio-Oss® Collagen in the buccal gap at immediate implants: a 6-month study in the dog. *Clinical Oral Implants Research*.

[22] Nemcovsky C. E., Artzi Z., Moses O., Gelernter I. (2000). Healing of dehiscence defects at delayed-immediate implant sites primarily closed by a rotated palatal flap following extraction. *International Journal of Oral & Maxillofacial Implants*.

[23] Nemcovsky C. E., Artzi Z. (2002). Comparative study of buccal dehiscence defects in immediate, delayed, and late maxillary implant placement with collagen membranes: clinical healing between placement and second-stage surgery. *Journal of Periodontology*.

[24] Elshahat A., Inoue N., Marti G., Safe I., Manson P., Vanderkolk C. (2005). Guided bone regeneration at the donor site of iliac bone grafts for future use as autogenous grafts. *Plastic and Reconstructive Surgery*.

[25] Zhang X., Awad H. A., O’Keefe R. J., Guldberg R. E., Schwarz E. M. (2008). A perspective: engineering periosteum for structural bone graft healing. *Clinical Orthopaedics and Related Research*.

[26] Rijal A. H., Dhami B., Pandey N., Aryal D. (2021). Prevalence of gingival pigmentation and its association with gingival biotype and skin colour. *Journal of Nepalese Society of Periodontology and Oral Implantology*.

[27] Rijal A. H., Dhami B., Ghimire P. (2022). Esthetic management of gingival hyperpigmentation with electrosurgery technique: a case report. *Journal of Chitwan Medical College*.

[28] Puisys A., Auzbikaviciute V., Vindasiute-Narbute E., Pranskunas M., Razukevicus D., Linkevicius T. (2022). Immediate implant placement vs. early implant treatment in the esthetic area. A 1-year randomized clinical trial. *Clinical Oral Implants Research*.

[29] Cooper L. F., Reside G. J., Raes F. (2014). Immediate provisionalization of dental implants placed in healed alveolar ridges and extraction sockets: a 5-year prospective evaluation. *International Journal of Oral & Maxillofacial Implants*.

